# Identification of Insider Trading in the Securities Market Based on Multi-task Deep Neural Network

**DOI:** 10.1155/2022/4874516

**Published:** 2022-04-22

**Authors:** Guofeng Li, Zuojuan Li, Zheji Wang, Ke Zhang

**Affiliations:** School of Statistics and Mathematics, Shandong University of Finance and Economics, Jinan 250014, China

## Abstract

Illegal insider trading identification is of great significance to the healthy development of the securities market. However, with the development of information technology, problems such as multidata sources and noise bring challenges to the insider trading identification work. Moreover, most of the current research on insider trading identification is based on single-task learning, which treats enterprises in different industries as a whole. This may ignore the differences between insider trading identification in different industries. In this article, we collect indicators from multiple sources to help regulators identify insider trading and then use information gain and correlation analysis to screen the indicators. Finally, we propose a multitask deep neural network with insider trading identification in different industries as different subtasks. The proposed model takes into account the correlations and differences between different tasks. Results of experiments show that compared with logistic, support vector machine, deep neural network, random forest, and extreme gradient boosting model, the proposed model can identify insider trading of enterprises in different industries more accurately and efficiently. This article provides new ideas for market regulators to maintain the order of the securities market through intelligent means.

## 1. Introduction

The securities market is an essential component of the financial market and has contributed significantly to the development of the world economy. However, due to asymmetric information, inadequate corporate governance structures, and poor regulatory mechanisms, illegal insider trading occurs frequently [1, 2]. Insider trading not only seriously undermines the fairness of securities trading, which has a huge impact on stock price volatility but also distorts the responsiveness of market prices to asset values and reduces the efficiency of asset allocation in the securities market [3, 4].

Governments have never ceased to combat and regulate insider trading, but the regulatory approach is mainly ex-post, with a significant lag [5]. How to strengthen the ex-ante prevention and timely stop enterprises from insider trading has become an important issue in maintaining market order. We should make full use of artificial intelligence technology to select reasonable regulatory indicators and establish scientific regulatory models to enhance the identification of insider trading.

Scholars select a variety of indicators to assist in the identification of insider trading. In general, stocks with insider trading have significantly nonzero excess returns [6]. The occurrence of insider trading increases the bid-ask spread of stocks and reduces the liquidity of the securities market [7]. Therefore, stock performance facilitates scholars to identify the presence of insider trading. Some scholars have studied insider trading in combination with corporate governance structures and found that the probability of insider trading occurring is related to the size of shareholders' rights. The greater the shareholders' rights, the greater the probability of insider trading occurring [8]. Therefore, banning insider trading helps maximize the value of the company and the interests of small-sized and medium-sized shareholders [9]. In addition to shareholding rights, executive compensation is also closely related to the occurrence of insider trading [10]. Companies with good governance can effectively limit the use of insider information by insider informants, thereby constraining the occurrence of insider trading [11]. In addition, the company's financial indices can help scholars determine whether insider trading has occurred [12]. Song and Li [13] selected 59 indicators to identify insider trading in terms of corporate governance, stock performance, and financial indices and found that seven indicators, such as the extraordinary cumulative return, were more effective. In addition to the above factors, the media's attention to listed companies can play an external monitoring intelligence, reducing the occurrence of irregular trading behaviors [14]. With the development of information and communication technologies, a large number of text documents are available on the web, such as media news reports about companies, which can be used as an important source of information for decision making [15]. We can use sentiment analysis to extract structured and informative knowledge from unstructured text and to classify text documents as positive, negative, and neutral [16]. Therefore, the use of news data can provide valuable information to decision makers for insider trading identification.

In early studies on insider trading identification models, scholars mostly used the event study approach to identify insider trading, that is, to identify the existence of insider trading by observing whether the market reacts to information in advance [17]. Some academics have also used models such as logistic and Probit to identify insider trading and have found these models to be more effective than the event study method [18]. With the development of artificial intelligence, machine learning algorithms are widely used to build robust and effective discriminatory and classification systems, such as random forests, fuzzy-rough nearest neighbor algorithms, and neural networks, and are widely used in economics, physics, medicine, and computer literature [19, 20]. With the help of machine learning, experts can identify insider trading more accurately. Now, methods such as decision trees, support vector machines, and neural networks have been gradually applied to insider trading identification [21, 22]. Zhang [23] used a support vector machine to identify insider trading, compared it with the traditional logistic method, and found that the support vector machine model predicted better. Deng et al. [21] proposed an identification method that integrates XGboost and NSGA-II for insider trading regulation.

An ex-ante identification model of insider trading based on indicators, such as stock performance, corporate governance, and financial indices, can track suspicious securities accounts promptly, but the selection of high-dimensional indicators is quite challenging. In addition, most studies on insider trading identification are based on single-task learning and treat enterprises in different industries as a whole. However, there are differences in the degree of development, business management activities, accounting, and financial management of different industries. These differences may have different effects on the insider trading activities of enterprises in different industries. If the data sets of different industries are modeled as a whole, the differences among insider trading of enterprises in different industries may be ignored; if the data sets of a single industry are modeled only, the correlations may be ignored. Single-task learning ignores possible relationships between different tasks, whereas multitask learning can take into account correlations and differences between tasks and improve the generalization ability of the model by exploiting the domain-specific information contained in the relevant tasks [24, 25]. Also, neural networks have certain advantages in dealing with nonlinear problems [26], so we propose a multitask deep neural network model for insider trading identification.

The contributions of our presented research are as follows: First, we collect data on stock performance, corporate governance, financial indices, and media coverage about enterprises using web scraping and text mining techniques, to enhance the model's ability to identify insider trading. Second, for the high-dimensional indicators, this research conducts feature selection based on information gain and correlation analysis. Third, taking the identification of insider trading in different industries as different subtasks, we propose a multitask deep neural network model to identify insider trading, and the proposed model is comprehensively compared with logistic, support vector machine (SVM), deep neural network (DNN), random forest (RF), extreme gradient boosting (XGBoost) models.

## 2. Methods

### 2.1. Indicators Screening Based on Information Gain and Correlation Analysis

**Figure 1:**
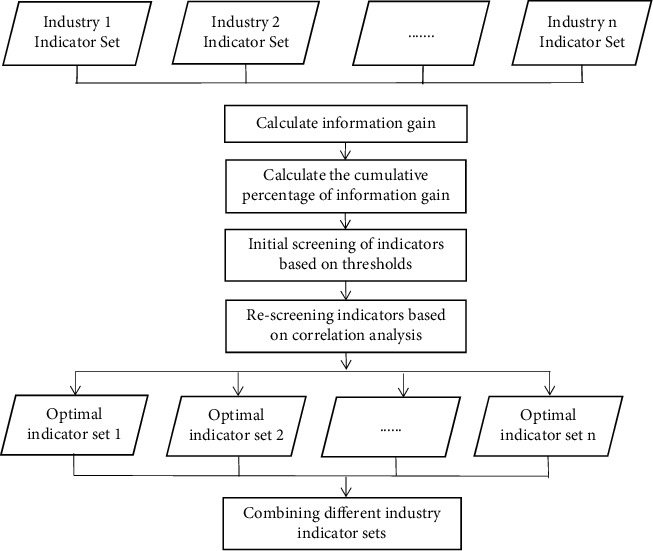
Flow chart of indicators screening.

Information gain has a strong performance in indicator variable selection [27]. To improve the operational efficiency and performance of the model, for the high-dimensional indicators, the information gain method is used to initially screen out the indicators with strong discriminatory ability, and then, correlation analysis is performed to further optimize the indicator set. The specific steps are as follows:

Step 1: Calculating the information gain for indicator *A*_*i*_: The information gain of indicator *A*_*i*_ is defined as Gain (*A*_*i*_), it is calculated based on information entropy and conditional entropy. The higher the information gain value of the indicator, the more classified information it carries, indicating that the indicator is more capable of distinguishing companies with insider trading.
Step 2: Calculating the percentage of information gain for indicator *A*_*i*_: Let *r*(*A*_*i*_) be the percentage of information gain for indicator *A*_*i*_, *θ* be the number of indicators, then
  (1) $$\begin{array}{c} r\left(A_{i}\right)=\frac{\text{Gain}\left(A_{i}\right)}{\sum_{i=1}^{\theta }\text{Gain}\left(A_{i}\right)}. \end{array}$$
Step 3: Calculating the cumulative percentage of information gain: The information gain percentage *r*(*A*_*i*_) are sorted from largest to smallest, noted as *r*_(1)_, *r*_(2)_,... *r*_(*θ*)_. Let *R*_*p*_ be the cumulative percentage of information gain for the first *p* indicators, then we have
  (2) $$\begin{array}{c} R_{p}=\overset{p}{\underset{i=1}{\sum }}r_{\left(i\right)}. \end{array}$$
The accumulation is stopped when *R*_*p*_ reached the threshold *K*, the corresponding *p* indicators are retained. In this research, the threshold *K* is chosen as 60%, 70%, and 80% to comparative analysis in subsequent experiments.
Step 4: Performing correlation analysis for the retained indicators: Among the pairs of indicators with high correlation, the indicators with small information gain values are deleted to avoid the duplication of information reflected between indicators.
Let *r*_kj_ denotes the correlation coefficient of the *k*th indicator with the *j*th indicator; *α*_ik_ denotes the standardized data of the *k*th indicator of the *i*th stock; $\bar{\alpha_{k}}$ denotes the mean of the standardized data of the *k*th indicator; *α*_ij_ denotes the standardized data of the *j*th indicator of the *i*th stock; $\bar{\alpha_{j}}$ denotes the mean of the standardized data of the *j*th indicator. Then,
  (3) $$\begin{array}{c} r_{\text{kj}}=\frac{\sum_{i=1}^{n}\left(\alpha_{\text{ik}}-\bar{\alpha_{k}}\right)\left(\alpha_{\text{ij}}-\bar{\alpha_{j}}\right)}{\sqrt{\sum_{i=1}^{n}{\left(\alpha_{\text{ik}}-\bar{\alpha_{k}}\right)}^{2}\sum_{i=1}^{n}{\left(\alpha_{\text{ij}}-\bar{\alpha_{j}}\right)}^{2}}}. \end{array}$$
A larger value of *r*_*kj*_ indicates a greater correlation between the *k*th indicator and the *j*th indicator.
Step 5: The final set of indicators is constructed by taking the ensemble of the screened indicators from different industries. The calculation process is shown in Figure 1.

### 2.2. Multitask Deep Neural Network

**Figure 2:**
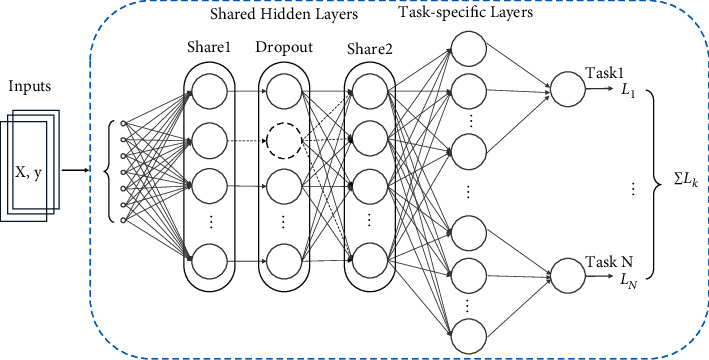
Identification of insider trading based on MTL-DNN.

Multitask learning, a promising branch of machine learning, has been widely used in computer vision, natural language processing, disease prediction [28–32], and other fields. We propose a multitask deep neural network model to identify insider trading, as shown in Figure 2. It is applied by sharing the hidden layers across all tasks while keeping several task-specific output layers to ensure the uniqueness of each task. Hard parameter sharing greatly reduces the risk of overfitting the model [25].

The model consists of a data input layer, two shared hidden layers, and task-specific output layers. Given that deep neural networks are slow to learn and prone to overfitting, we use the dropout method for structural refinement of shared layer 1 to enhance the generalization of the model. That is, a certain percentage of hidden layer nodes are randomly discarded during forwarding propagation, as shown in the circle marked by the dashed line in Figure 2. The forward propagation of the network is calculated as

(4) $$\begin{array}{c} y^{\left[l\right]}=g_{l}\left(b_{l}+y^{\left[l-1\right]}w\right), \end{array}$$

where *y*^[*l*]^ is the output of the *l*th hidden layer, *y*^[*l* − 1]^  is the input of the *l*th hidden layer, *w* and  *b*_*l*_ are the weight and bias matrices, respectively, and *g*_*l*_ is the Sigmoid activation function. Then, multitask loss functions are jointly optimized, as shown in formula (5).

(5) $$\begin{array}{c} L=\overset{N}{\underset{k=1}{\sum }}L_{k}=\overset{N}{\underset{k=1}{\sum }}f\left({\hat{y}}_{k},y_{k}\right), \end{array}$$

where *f*(.) is the error function that measures the difference between the predicted and true values of the model for the *k*th task. In this article, a cross-entropy loss function is chosen for the classification problem.

In the error back-propagation process, we use the Adam optimization algorithm to update the weights, which has the characteristics of fast and stable convergence [33]. Assuming that at moment *t*, the Adam algorithm is calculated as

(6) $$\begin{array}{c} m_{t}=\beta_{1}m_{t-1}+\left(1-\beta_{1}\right)g_{t},\;g_{t}=\nabla_{\left(w,b\right)}L_{k},\\ v_{t}=\beta_{2}v_{t-1}+\left(1-\beta_{2}\right)g_{t}^{2},\;g_{t}^{2}=g_{t}⊙g_{t},\\ \hat{m_{t}}=\frac{m_{t}}{1-\beta_{1}^{t}},\\ \hat{v_{t}}=\frac{v_{t}}{1-\beta_{2}^{t}}\;,\\ {\left(W,b\right)}_{t+1}\leftarrow {\left(W,b\right)}_{t}-\frac{\eta }{\sqrt{{\hat{v}}_{t}}+\varepsilon }{\hat{m}}_{t}, \end{array}$$

where *η* is the learning rate, and *m*_*t*_ and *v*_*t*_ are first moment estimate and second raw moment estimate, respectively. *β*_1 _^*t*^ and *β*_2_^*t*^ are exponential decay rates for the two moment estimates.

Multitask learning has the advantages of data amplification, eavesdropping, offsetting some of the noise, and preventing overfitting, especially in the case of small sample sizes [25]. Multitask deep neural networks improve the identification of enterprise insider trading by building shared hidden layers and task-specific layers that take into account the correlation and difference of different tasks.

To evaluate the classification effect of the model, we select four common statistical indicators, including accuracy, recall, F1-score, and AUC. Accuracy represents the proportion of the observations that are predicted correctly. The recall rate represents the probability that a true positive sample is predicted as a positive sample; F1-score is the weighted average of precision and recall. The AUC is the area under the ROC curve; the higher the area under the curve, the better the predictions are.  Table 1 shows the confusion matrix, and the equations for each evaluation indicator are shown in formulas (7)–(9).


(7) $$\begin{array}{c} \text{Accuracy}=\frac{\text{TP}+\text{TN}}{\text{TP}+\text{TN}+\text{FP}+{\text{FN}}^{\prime }} \end{array}$$

(8) $$\begin{array}{c} \text{Recall}=\frac{\text{TP}}{\text{TP}+{\text{FN}}^{\prime }} \end{array}$$

(9) $$\begin{array}{c} F1-\text{score}=\frac{2\times \text{Precision}\times \text{Recall}}{\text{Precision}+\text{Recall}}\;. \end{array}$$

## 3. Data and Indicators

The research takes the Chinese securities market as an example. The samples contain two categories: one is positive samples, i.e., companies in China's manufacturing industry that had insider trading and have been notified and punished by the regulator from 2001–2019; the other is negative samples, i.e., companies that have had the same type of significant events and have not been punished by the regulator. Positive samples match negative samples, and the matching principles are as follows: first, the same exchange; second, the same industry; third, the same year of significant events; fourth, the same company size.

Based on the number of manufacturing subindustries and the number of enterprises with insider trading, we select five manufacturing subindustries, including computer communication and electronic equipment manufacturing, pharmaceutical manufacturing, chemical materials and products manufacturing, electrical machinery and equipment manufacturing, and special equipment manufacturing. The data category ratio for positive and negative samples is 1 : 3.5. To address the problem of small sample data and category imbalance, we use the SMOTE method [34] to oversample the positive samples in the experiment. Finally, the data set contains 998 samples, including 499 negative samples and 499 positive samples.

Through literature survey and theoretical research, we divide the indicators of insider trading identification into four categories. One is the stock performance, which is the most intuitive indicator to identify insider trading. The second is the corporate governance. Generally, people involved in insider trading are mostly the controlling shareholders and executives of the company, mainly because such people tend to have the easiest access to insider information. The third is the financial indices, which is a relative indicator for enterprises to summarize and evaluate their financial status and operating results. Fourth, the media coverage; media coverage can perform an external monitoring function that may reduce the incidence of irregular trading practices. We use text sentiment analysis based on sentiment lexicon to process news data [35]. We found that 71% of the insider trading occurred within three months before the announcement of the material information after counting the sample data. Therefore, we select the media coverage of relevant companies in the three months before the date of the significant information announcement. Finally, we obtain a total of about 40,000 news reports. The indicators mentioned above are mainly from the RESSET database and the CSMAR database, and partly through web scraping. Finally, we initially select 60 indicators. The descriptions of the indicators are shown in Table 2.

## 4. Experimental Results

### 4.1. Feature Selection Based on Information Gain and Correlation Analysis

In this article, we collect indicators from multiple sources and further filtered the indicators to improve the model performance. We use information gain and correlation analysis to filter indicators for each of the five industry data sets according to the process in Figure 1, and finally, take the ensemble to construct the indicator set. The indicators are selected according to the threshold values of 60%, 70%, and 80%, respectively, and comparative analysis is performed to select the most appropriate indicator set.

**Figure 3:**
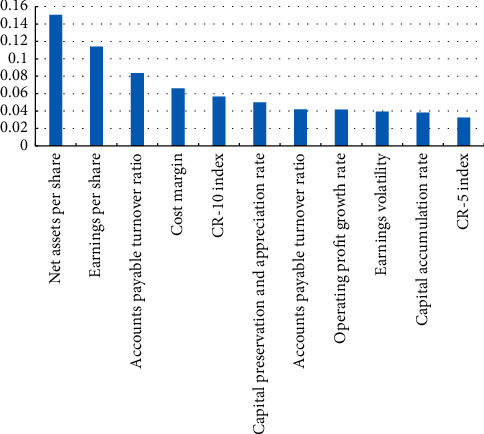
Information gain ranking of chemical manufacturing indicators.

Taking 70% as an example, Figure 3 shows the results of the rank of the information gain of chemical raw materials and products manufacturing, and 11 indicators with the top 70% of cumulative information gain are selected. Two pairs of indicators with strong correlation are obtained by correlation analysis, as is shown in Table 3, and then, we eliminate indicators with small information gain values in each pair, leaving 9 indicators. Similar operations are performed for the other four types of industries, and finally, the selected indicators from the five industry data sets are combined to obtain a total indicator set containing 19 indicators, as is shown in Table 4.

### 4.2. Comparative Analysis of Different Indicator Sets

Based on different thresholds, we construct three different sets of indicators and select the best one by comparative analysis. The data are randomly divided into training and testing sets in the ratio of 70% : 30%. We take the average of the ten rounds of test results. In addition, we mainly select DNN and MTL-DNN methods for comparative analysis, and the results are shown in Table 5. The dropout rate is 0.4. Adam optimization algorithm learning rate *η* = 0.001, exponential decay rate *β*_1_ = 0.9, and *β*_2_ = 0.9999.

In Table 5, Pool-DNN denotes the modeling results based on the overall industry data set by DNN; STL-DNN denotes the mean value of the results after modeling separately for five single industry data set by DNN. The evaluation indicators such as accuracy, recall, and AUC are the best when the threshold is 70%. So we finally select the set of indicators corresponding to the threshold value of 70% and use it when comparing the models below. It can be seen that the performance of the model was further improved using information gain and correlation analysis for index screening. The model performance of MTL-DNN is better than DNN under several different indicator sets, indicating that the model constructed in this article has strong insider trading identification capability and strong robustness.

### 4.3. Comparative Analysis of Different Models

To validate the model's effectiveness, this article compares the proposed model with methods, such as logistic, SVM, DNN, RF, and XGBoost; Table 6 reports the results. Among several methods, the logistic has the lowest values of indicators, such as F1-score and recall. The model proposed in this article performs the best, with better evaluation indicators, such as accuracy, recall, F1-score, and AUC.

Given that the main objective of this research is to identify companies with insider trading, we focus on the recall rate. The recall rate means the percentage of companies with insider trading that are accurately identified. Table 6 shows that the recall rate of the MTL-DNN model is 90.6%, which is a significant improvement of 7.6% and 6.5% over STL-DNN and Pool-DNN. Compared with Logistic, SVM, DNN, RF, and XGBoost, the model proposed has better evaluation metrics, such as accuracy, recall, F1-score, and AUC. It indicates that the model proposed in this article was more accurate in insider trading identification.

### 4.4. Comparative Analysis of Models with Different Number of Industries

In this article, we select five subindustries of the manufacturing industry for the experiment. To test the effectiveness of the model constructed in this article after adding more other industries as subtasks, this section further compares the indexes of the MTL-DNN model with a different number of tasks. The number of tasks is 2, i.e., we randomly select two as tasks from five industry data sets, there are ten combinations in total, and then take the average value of the index results, and so forth.

**Figure 4:**
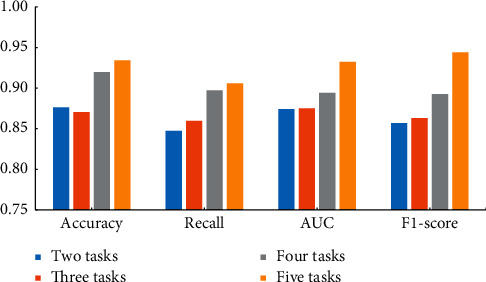
Comparison of MTL-DNN with different number of tasks.

As is shown in Figure 4, with the increase of the number of tasks, the evaluation indexes (i.e., F1-score, recall rate) of the MTL-DNN model show an increasing trend. It demonstrates that by adding more subindustries, more information can be shared between different tasks, which may yield better results. Therefore, the model proposed in this article can add more industries and has good scalability.

## 5. Conclusions

This article utilizes data from multiple sources to improve the research validity. For high-dimensional indicators, this article uses information gain and correlation analysis to filter indicators. Taking the identification of insider trading in different industries as different subtasks, this article constructs a multitask deep neural network model, which considers the correlation and heterogeneity among insider trading activities in different industries. Compared with traditional machine learning methods, such as Logistic, SVM, RF, and XGBoost, the model proposed in this article performs better with higher values of evaluation metrics, such as accuracy, recall, and AUC. This research is helpful to market regulators to improve their supervision accuracy and efficiency on insider trading identification. Moreover, it is of great significance to provide timely supervision by intelligent means.

The data set constructed in this article can be further enriched with application scenarios in the future to serve other fields, such as enterprise credit system construction. Second, although the multitask model constructed in this article is only applied to some of the industries in the experiment, it can be easily extended to more industries. Finally, we can use the model proposed in this article to try to solve other aspects of the companies' identification problem for further research.

## Figures and Tables

**Table 1:** Confusion matrix.

Class		Predicted class	
		1	0
Actual	1	TP (True positives)	FN (false negatives)
Class	0	FP (false positives)	TN (true negatives)

**Table 2:** Description of relevant indicators.

Indicator category	Indicator dimension	Indicator name	Indicator description
Stock performance	Earning	Average daily return	Average daily return of 30 trading days before the material information announcement date
		Yield amplitude	Difference between the maximum return and minimum of 30 trading days before the material information announcement date
		Cumulative excess return	Accumulation excess return of the 30 trading days before the material information announcement date
		Cumulative excess return amplitude	Difference between the maximum cumulative excess return and the minimum of the 30 trading days before the material information announcement date
	Liquidity	Relative turnover rate	The ratio of the sample shares actual turnover and the reference turnover from T-150 days to T-30 days
		Relative spread	The ratio of the actual spread and the average daily spread from T-150 days to T-30 days
		Relative trading volume	The ratio of the actual trading volume and the average daily trading volume from T-150 days to T-30 days
		Relative illiquidity ratio	The ratio of the actual noncurrent ratio and the average daily noncurrent ratio from T-150 days to T-30 days
		Relative amplitude	The ratio of the actual amplitude and the average daily amplitude from T-150 days to T-30 days
	Volatility	Earnings volatility	The standard deviation of the sample stocks returns for the 30 trading days before the information release
	Riskiness	BETA	Average daily beta of 30 trading days before the material information announcement date

Corporate governance	Meeting status	The meeting times of the board of directors, the meeting times of the board of supervisors, etc.	
	Executive information	The first three directors' total compensation, the first three executives' total compensation, independent director number, number of directors holding shares, shareholding ratio of the supervisory board, etc.	
	Shareholding status	CR-5 index, CR-10 index, *Z* index, Herfindahl-5 index, shareholding ratio of the largest shareholder, shareholding ratio of top five shareholders, etc.	

Financial indices	Solvency	Current ratio, quick ratio, cash ratio, asset debt ratio, interest coverage ratio, property right ratio	
	Operating capacity	Inventory turnover, current asset turnover, fixed asset turnover ratio, total asset turnover ratio, accounts payable turnover ratio	
	Profitability	Net assets per share, earnings per share, operating margin, cost margin, return on assets, etc.	
	Growth capacity	Total asset growth rate, operating revenue growth rate, operating profit growth rate, capital preservation and appreciation rate, capital accumulation rate, etc.	

Media coverage	Media attention	The number of news reports in the 3 months before the material information announcement date	
	Negative media sentiment	The sentiment score of each news report in the 3 months before material information announcement date is calculated, and then, we calculate the proportion of reports with negative sentiment scores	

Insider trading	Insider trading	A binary variable that takes value of 1 if insider trading occurred and 0 otherwise	

**Table 3:** Correlation analysis of chemical manufacturing indicators.

Number	Retained indicators		Deleted indicators		Correlation coefficient
	Indicators	Information gain	Indicators	Information gain	
1	CR-10 index	0.056402	CR-5 index	0.032328	0.997
2	Capital preservation and appreciation rate	0.050051	Capital accumulation rate	0.038245	0.995

**Table 4:** The set of insider trading indicators at a threshold of 70%.

Indicator category	Indicator dimension	Indicator name
Stock performance	Earning	Average daily return
		Cumulative excess return
	Volatility	Earnings volatility
	Liquidity	Relative spread
	Riskiness	BETA

Corporate governance	Meeting status	The meeting times of the board of supervisors
	Shareholding status	CR-10 index
		Shareholding ratio of the largest shareholder

Media coverage	Media coverage	Negative media sentiment

Financial indices	Growth capacity	Operating profit growth rate
		Capital preservation and appreciation rate
	Operating capacity	Inventory turnover ratio
		Fixed assets turnover ratio
		Accounts payable turnover ratio
	Profitability	Earnings per share
		Net assets per share
		Cost margin
	Solvency	Gearing ratio
		Equity ratio

**Table 5:** Comparison of modeling effects for different index sets.

Threshold	60%			70%			80%		
Methods	STL-DNN	Pool-DNN	MTL-DNN	STL-DNN	Pool-DNN	MTL-DNN	STL-DNN	Pool-DNN	MTL-DNN
Accuracy	0.871	0.883	0.884	0.818	0.901	0.934	0.701	0.741	0.888
Recall	0.883	0.885	0.889	0.830	0.841	0.906	0.803	0.761	0.889
AUC	0.849	0.884	0.861	0.828	0.907	0.932	0.798	0.883	0.890
F1-score	0.893	0.881	0.897	0.887	0.898	0.944	0.809	0.879	0.886

**Table 6:** Comparison of multiple models in insider trading identification.

Methods		Logistic		SVM		RF		XGBoost		DNN		
		Pool	STL	Pool	STL	Pool	STL	Pool	STL	Pool	STL	MTL
Accuracy	Industry1	—	0.837	—	0.811	—	0.963	—	0.974	—	0.889	0.868
	Industry2	—	0.849	—	0.939	—	0.849	—	0.859	—	0.846	0.969
	Industry3	—	0.875	—	0.854	—	0.965	—	0.931	—	0.667	0.909
	Industry4	—	0.794	—	0.830	—	0.766	—	0.765	—	0.788	0.960
	Industry5	—	0.800	—	0.789	—	0.903	—	0.974	—	0.902	0.966
	Average	0.856	0.831	0.813	0.845	0.901	0.889	0.874	0.900	0.901	0.818	0.934

Recall	Industry1	—	0.615	—	0.732	—	0.867	—	0.946	—	0.889	0.871
	Industry2	—	0.529	—	0.861	—	0.652	—	0.582	—	0.861	0.911
	Industry3	—	0.750	—	0.940	—	0.843	—	0.615	—	0.708	0.893
	Industry4	—	0.511	—	0.760	—	0.538	—	0.500	—	0.789	0.939
	Industry5	—	0.600	—	0.952	—	0.819	—	0.964	—	0.903	0.917
	Average	0.682	0.601	0.858	0.849	0.758	0.744	0.724	0.721	0.841	0.830	0.906

AUC	Industry1	—	0.782	—	0.771	—	0.914	—	0.973	—	0.892	0.871
	Industry2	—	0.868	—	0.890	—	0.734	—	0.869	—	0.868	0.967
	Industry3	—	0.920	—	0.750	—	0.909	—	0.977	—	0.685	0.896
	Industry4	—	0.769	—	0.915	—	0.651	—	0.690	—	0.790	0.960
	Industry5	—	0.726	—	0.722	—	0.911	—	0.987	—	0.902	0.966
	Average	0.763	0.813	0.791	0.810	0.879	0.824	0.902	0.899	0.907	0.828	0.932

*F*1-score	Industry1	—	0.667	—	0.826	—	0.836	—	0.972	—	0.930	0.878
	Industry2	—	0.546	—	0.872	—	0.634	—	0.650	—	0.949	0.969
	Industry3	—	0.692	—	0.836	—	0.906	—	0.762	—	0.874	0.973
	Industry4	—	0.632	—	0.867	—	0.557	—	0.533	—	0.763	0.958
	Industry5	—	0.643	—	0.936	—	0.825	—	0.947	—	0.921	0.945
	Average	0.677	0.636	0.814	0.867	0.789	0.752	0.846	0.773	0.898	0.887	0.944

## Data Availability

The data used to support the findings of the study can be obtained from the author upon request.
